# Alveolar and gingival necrosis resulting from chloroform overextrusion beyond the root canal: A case report

**DOI:** 10.1002/ccr3.9472

**Published:** 2024-10-29

**Authors:** Zahra Sadat Modarresi, Sahar Shafagh, Mehrzad Kaviani, Mojtaba Salehi Karizmeh

**Affiliations:** ^1^ Department of Oral and Maxillofacial Surgery and Craniomaxillofacial Research Center, Faculty of Dentistry Tehran University of Medical Sciences Tehran Iran; ^2^ Department of Endodontics Qazvin University of Medical Sciences Qazvin Iran

**Keywords:** case report, chloroform, gingival necrosis, gutta‐percha, root canal apex

## Abstract

Chloroform is among the most common solvents for root canal treatment. Despite its classification as a potential carcinogen, it is effective if used correctly. Inappropriate use can lead to severe complications, including persistent pain, gingival recession, and necessitating surgical intervention to resect necrotizing bone.

## 1 INTRODUCTION

It is essential to fully seal the root canal after cleaning and shaping to ensure the success of endodontic treatment.^1^, ^2^ However, during the process, there is a risk of gutta‐percha overextension, which can have negative consequences.^2^, ^3^ Overextension of materials beyond the apex can cause mechanical irritation and trigger an inflammatory reaction in the periapical region.^2^

Gutta‐percha is a commonly used material for root canal obturation. It can be easily removed in cases of endodontic failure or overextension beyond the apex.^4^ There are several methods to remove gutta‐percha from the root canal including thermal, mechanical, ultrasonic, and chemical solvents.^4^, ^5^, ^6^, ^7^, ^8^ When removing gutta‐percha, using only thermo‐mechanical techniques can lead to complications such as ledges, transportation, and broken instruments. The use of chemical solvents can reduce transportation by decreasing the force applied during negotiation.^8^, ^9^ Moreover, the use of solvents compared with no use of solvents can lead to less apically extruded debris and irrigant.^10^ Furthermore, The ProTaper Next (PTN) system, when used in conjunction with the martensitic nickel‐titanium hybrid (MNiTiH) instrument, demonstrates a superior ability to remove root canal filling materials. This combination is more effective and results in less apical debris extrusion compared to using the PTN system with chloroform. The use of solvents like chloroform in endodontic procedures helps dissolve and facilitate the removal of gutta‐percha and other root canal sealers. Therefore, by using MNiTiH instruments, which are designed to enhance flexibility and cutting efficiency, cleaner canal spaces with minimized risk of debris extrusion and improving the overall outcomes are achieved.^11^ However, one of the main concerns when using solvents is their potential to be extruded beyond the apex of the canal during gutta‐percha removal.^8^, ^12^ Extruding solvents can cause irritation and impact the healing process of periapical tissue.^8^ Chloroform is frequently used as an inorganic solvent to quickly soften gutta‐percha for removal.^4^, ^5^, ^6^, ^13^ It has been proven to be effective and safe as a gutta‐percha solvent.^4^, ^14^ However, there have been reported side effects of exposure to chloroform.^4^, ^15^ The International Agency for Research on Cancer has classified chloroform as a group 2B carcinogen, which means there is insufficient evidence to prove its carcinogenicity in humans, but there is enough evidence to indicate that it is a carcinogen in animals.^4^, ^6^

There are some reports of soft tissue necrosis following accidental chloroform injection instead of local anesthesia or for endodontic retreatment.^4^, ^5^ Overall, the soft tissue necrosis after the chloroform injection in dental procedures are important complication that should be considered. Hence, the documentation and reporting of such cases can serve as a valuable educational tool for dentists. Herein, we reported a case of alveolar and gingival necrosis caused by over extrusion of chloroform beyond the root canal apex.

## 2 CASE HISTORY/EXAMINATION

A 48‐year‐old woman, without any prior health issues, was referred to our department by a general practitioner. The treatment plan was to place a dental prosthesis in the form of a bridge in the upper anterior region. The left upper lateral incisor was intended to be one of the bridge abutments. However, during the procedure, the tooth became exposed and required endodontic treatment. Chloroform was used during the procedure, which resulted in overextension and subsequent necrosis of the bone.

**FIGURE 1:**
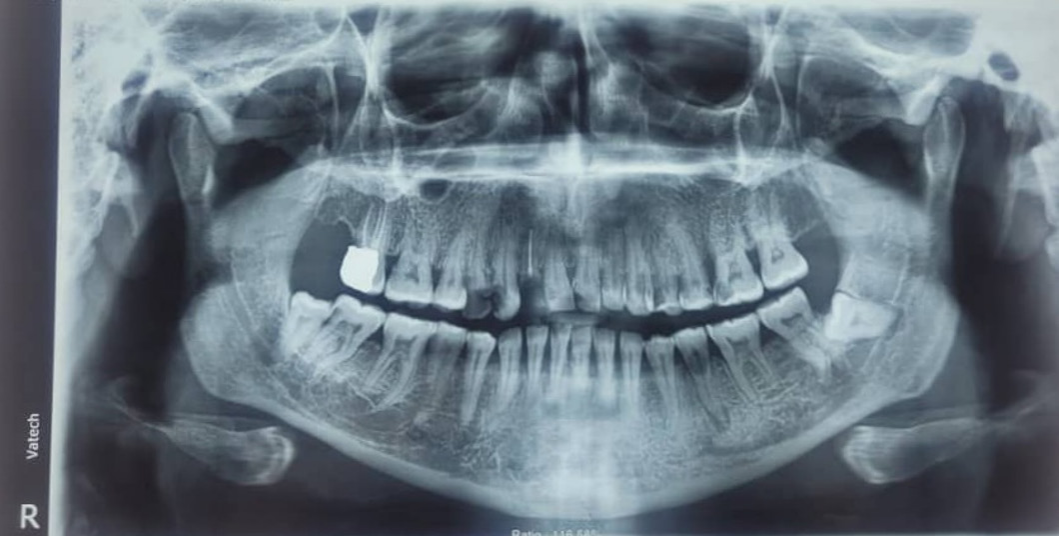
Baseline radiography before the root canal treatment.

**FIGURE 2:**
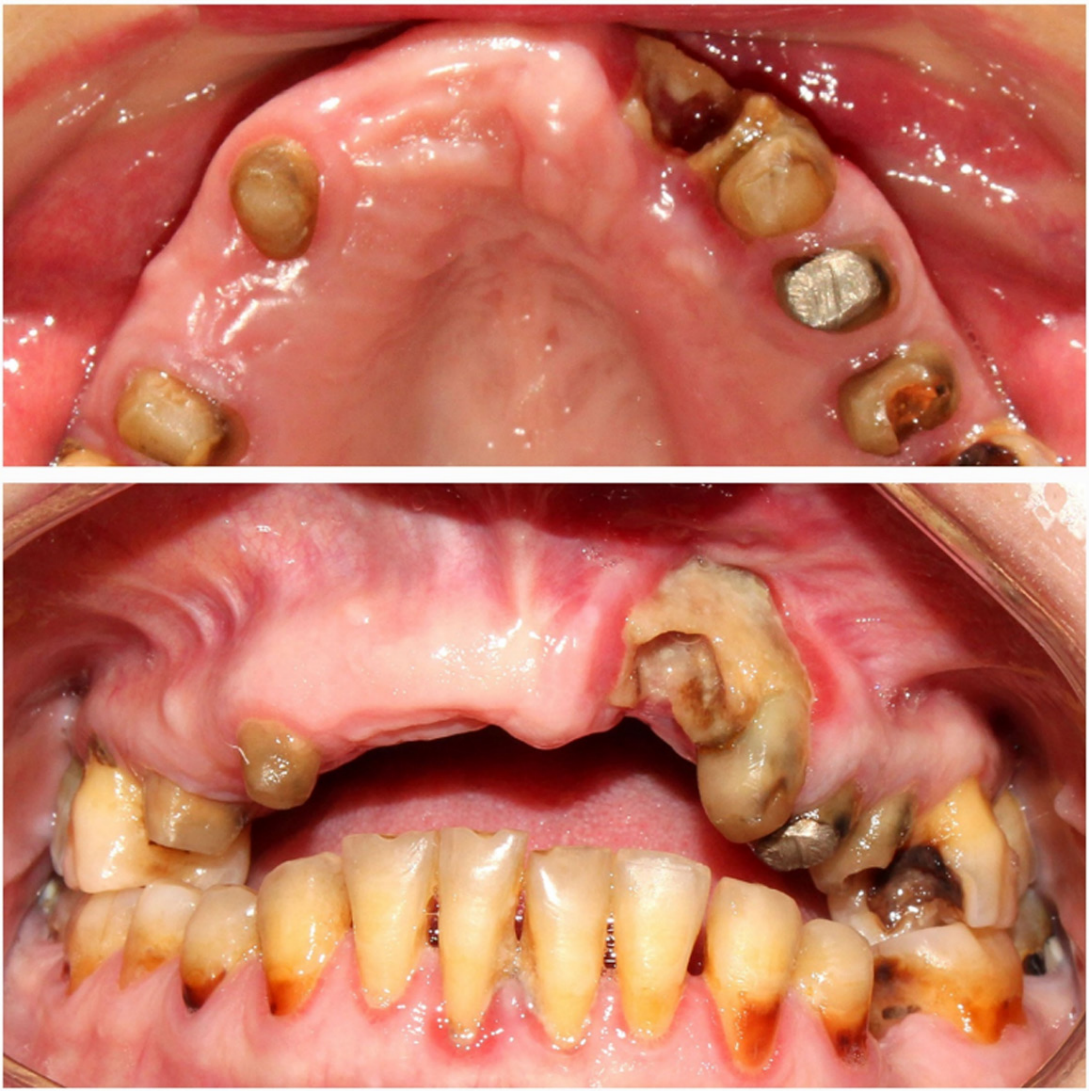
Gingival recession and alveolar bone necrosis after the extraction of the tooth.

The initial radiograph (Figure 1) was taken prior to this treatment. It is important to note that between the time the baseline radiograph was taken and the recent endodontic treatment, the upper central incisors, the right upper lateral incisor, and the right upper first premolar were extracted due to caries (Figure 2). However, no new radiograph was taken immediately before the endodontic treatment of the left upper lateral incisor.

## 3 METHODS

After overfilling the gutta‐percha, the dentist immediately decided to remove it. Despite the short time frame, the practitioner used chloroform as a solvent due to significant resistance encountered with files alone. Chloroform was employed to remove gutta‐percha and excess material from the canal. However, because of high injection pressure, some chloroform extruded beyond the apex. This incident caused sudden and severe pain in the patient. The gutta‐percha was removed, and the tooth was obturated again. The patient was discharged with analgesics, antibiotics, and dexamethasone.

**FIGURE 3:**
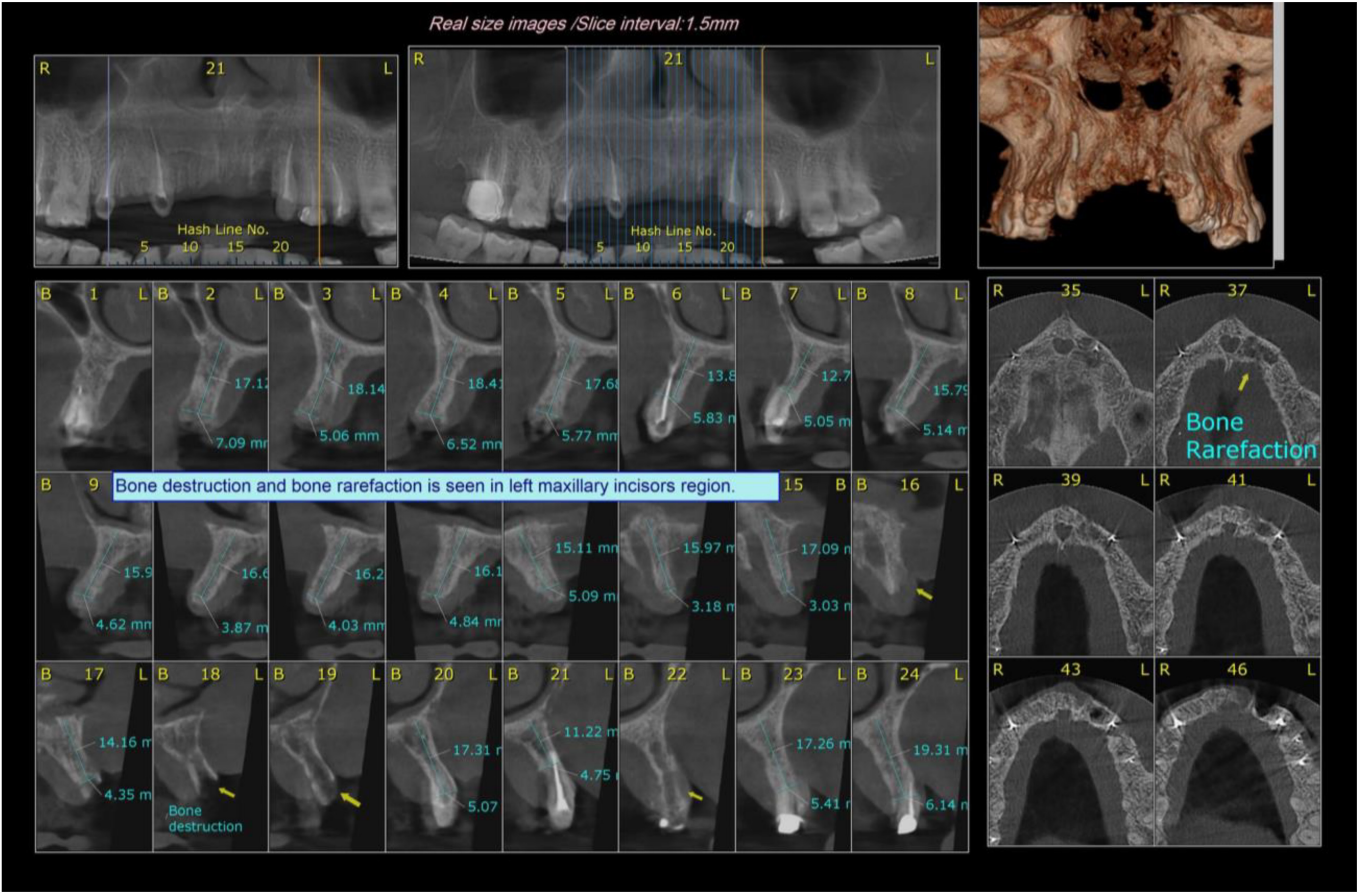
Cone beam computed tomography shows bone destruction in the left maxillary incisor region.

**FIGURE 4:**
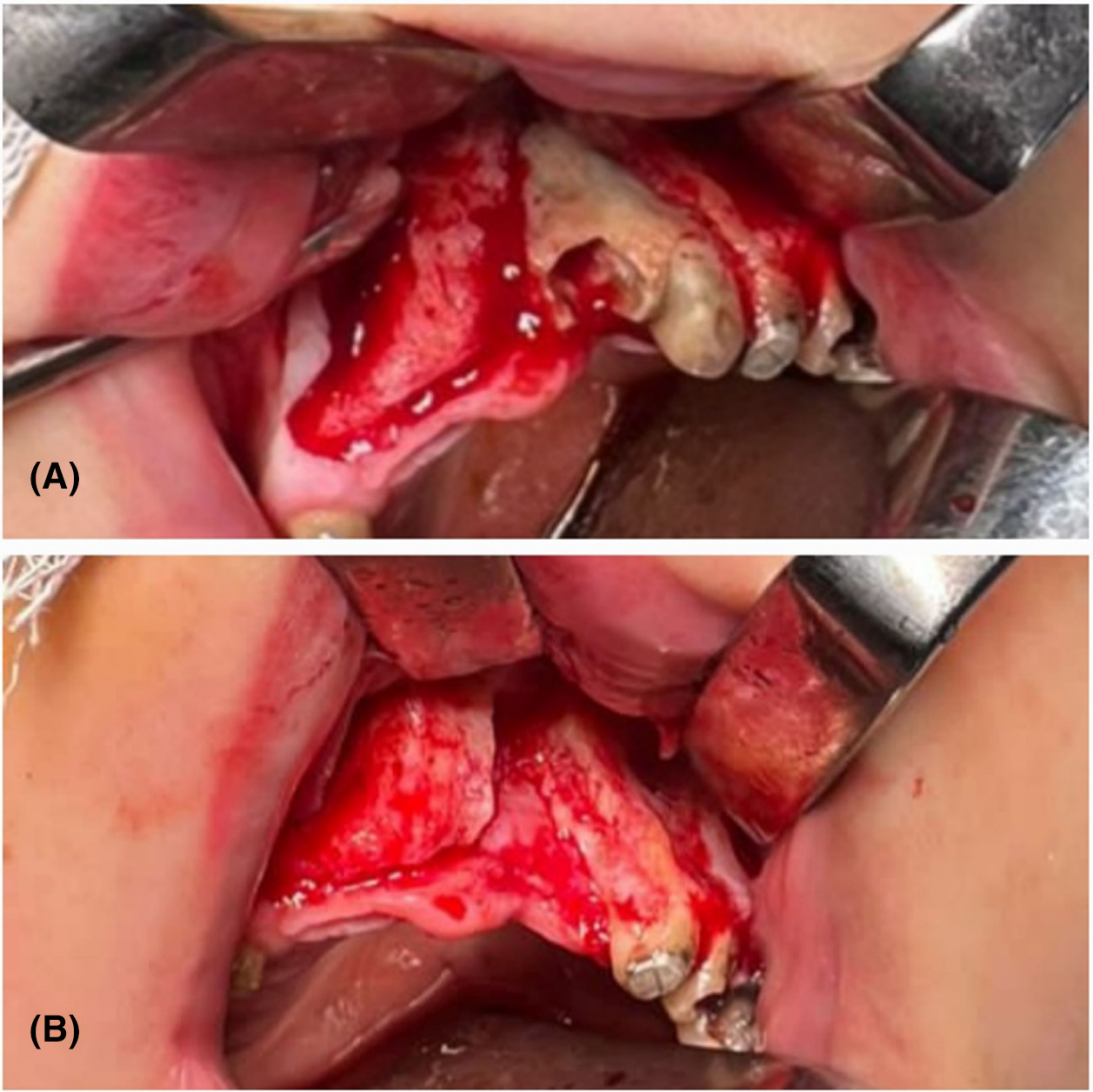
(A) Alveolar bone necrosis, (B) Necrotic bone resection in the operating room.

The patient continued to experience pain and the tooth became mobile. Following 1 week, the tooth had to be extracted. Gingival recession and exposure of the surrounding alveolar bone occurred after the extraction of the tooth. The patient was referred to a periodontist who attempted to treat the gingival recession with a free gingival graft, while it was unsuccessful. Finally, the patient was referred to our department to be examined by a maxillofacial surgeon. A preoperative cone beam computed tomography was performed to evaluate the bone condition (Figure 3). She was moved to the operating room to resect the necroting bone under the general anesthesia (Figure 4). A 2% lidocaine solution with 1:100,000 epinephrine was administered. A sulcular flap in the areas above the tooth and a crestal flap in the anterior maxilla with bilateral releasing incisions were performed. The necrotic alveolar bone was completely resected down to bleeding bone. The area was irrigated, the flap was repositioned, and sutured with 4–0 Vicryl sutures. She was hospitalized in the ward for 1 day and received cefazolin 1 g every 6 h, dexamethasone 8 g every 8 h, analgesics, and serum therapy. She was discharged with prescriptions for analgesics and antibiotics, including acetaminophen 500 mg every 8 h for 5 days, chlorhexidine 0.2% mouthwash every 12 h for 1 week, and cephalexin 500 mg every 6 h for 1 week.

## 4 CONCLUSION AND RESULTS

**FIGURE 5:**
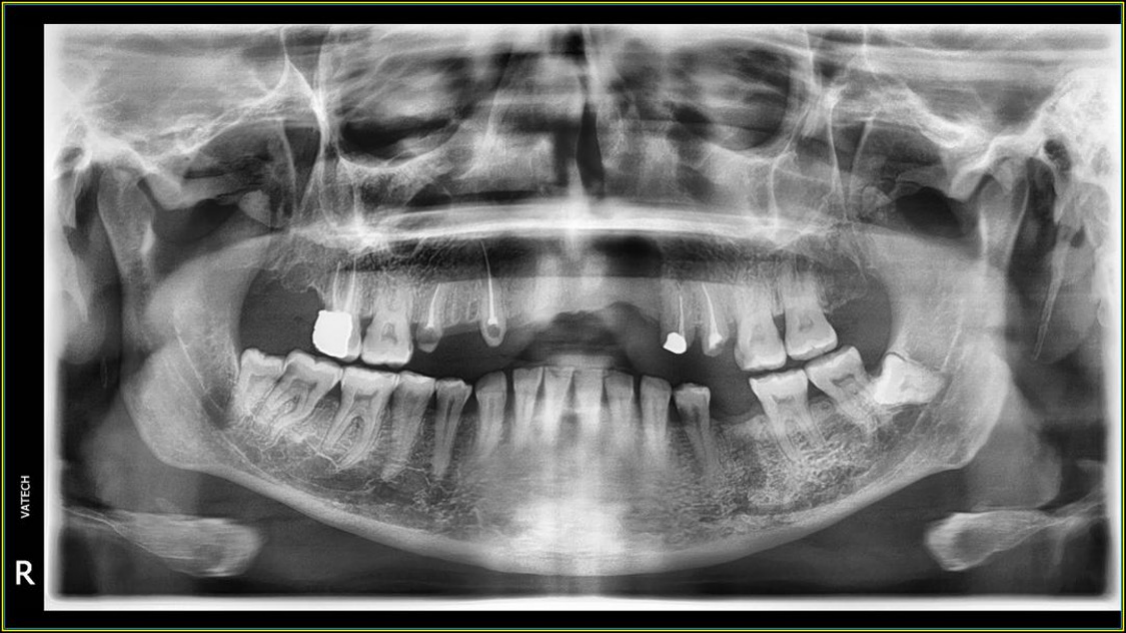
Postoperative radiography.

**FIGURE 6:**
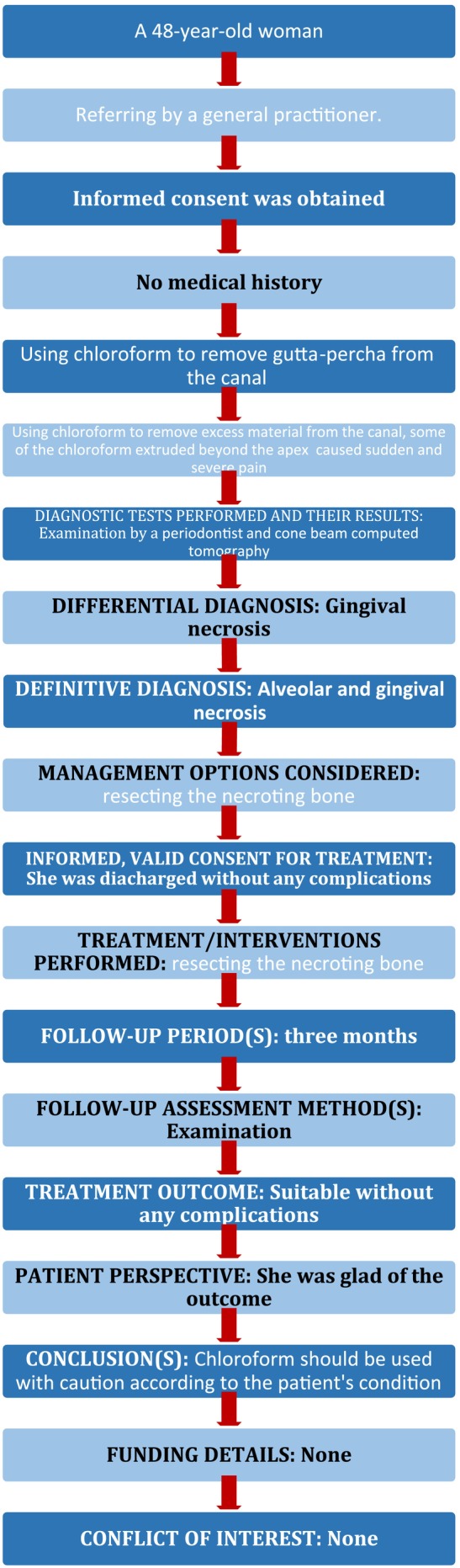
PRICE 2020 flowchart.

A postoperative radiography was conducted to evaluate the conditions following the surgical resection of the necrotizing bone (Figure 5). She was referred to her practitioner for follow‐up and treatment continuation. During the follow‐up telephone call, no complications were reported. However, the patient did not intend to continue her treatment. Consequently, no follow‐up intraoral photographs, periapical radiographs, or cone beam computed tomography images were obtained. The PRICE flowchart is provided in Figure 6.

## 5 DISCUSSION

This case report presented a situation where chloroform leaked into the periapical tissue during the retreatment of a tooth with gutta‐percha. This led to the necrosis of the alveolar bone and loss of the tooth.

The primary goal of using solvents is to overcome the challenge of accessing and effectively removing condensed obturator material, especially in curved root canal regions where perforation risk is high. Solvents soften or dissolve the obturator material, making it to remove without compromising the integrity of the root canal.^16^ Numerous solvents, including chloroform, eucalyptol, orange oil, tetrachloroethylene (Endosolv), and xylene, are accessible; however, none completely fulfill the criteria of an ideal solvent. Ideally, solvents should be non‐toxic and non‐carcinogenic to both patients and clinicians, effectively soften gutta‐percha, remain viable for a sufficient duration, and be cost‐effective.^17^ It should be noted that all solvents used for gutta‐percha retreatment have some level of toxicity.^18^ Chloroform, being one of the most used solvents, can cause toxicity when it comes in contact with periapical tissue.^6^ However, the use of chloroform in small and controlled amounts does not have the risk of toxicity.^12^, ^19^ Chloroform can have negative effects on periapical tissue in several conditions, including root resorption, open apex, perforation, and destroyed apical constriction as reported in this case. Verma et al. also reported a case where chloroform was mistakenly injected instead of local anesthetic. The patient felt sharp pain, and when the injection was stopped, numbness and tissue necrosis occurred.^5^ Furthermore, in another case, a chemical burn of the skin while using chloroform occurred which was likely due to dropping on the patient skin, whereas gingiva and oral mucosa were intact.^20^ Accordingly, Taghavi Zenouz and colleagues reported a necrotic ulcer on the hard palate due to injecting chloroform into the root canal to relieve pain without using a rubber dam. This highlights the significance of proper isolation during endodontic procedures.^21^ In the case report published by Mohammadzadeh Akhlaghi et al., there was a tissue necrosis caused by chloroform following a perforation that occurred during access cavity preparation in an addicted patient. In this case, the patient did not experience pain, which was thought to be due to his addiction.^4^ However, it should be noted that there are some benefits associated with chloroform, such as significantly improved removal of gutta‐percha using mechanical files, advantages in cases where mechanical methods were ineffective in retrieving gutta‐percha or faced difficulty in removing filling material, and reduced time required for retreatment.^22^ Using solvents during the process of removing root fillings resulted in fewer instances of debris and irrigant being extruded apically compared to not using solvents. Chloroform showed a notably higher extrusion of debris compared to orange oil and turpentine oil, with statistical significance.^10^ In general, regardless of whether the clinician employs manual or mechanical instrumentation techniques, the utilization of solvents during obturation removal may present drawbacks in terms of root canal cleanliness. Thus, it should only be considered if accessing the previous working length proves difficult without its application.^22^ Furthermore, research investigating the efficacy of solvents in removing root filling materials often fails to directly compare various solvents. This lack of comparative analysis makes it challenging, and potentially misleading, to draw conclusions regarding the effectiveness of any one solvent over others.^22^ The study was one of the examples of carelessly using chloroform and its complications. Future studies that directly compare the performance and impacts of different solvents in efficiently removing gutta‐percha is recommended.

Generally, the findings underscore the importance of considering of any intense or severe pains that patients may experience when using chloroform. The usage of chloroform should be done with caution, in small amounts, and it is advised to avoid locking needles and applying excessive pressure during its use. In addition, retreatment techniques without solvents can be utilized in the apical part, which includes rotary systems or hand files, to reduce possible risks.^23^, ^24^, ^25^ It is suggested that the use of solvents to remove the overextended gutta‐percha would make it soften and more difficult to remove. Hence, this aspect must be taken into account while doing retreatments.^26^

One of the limitations of the study is that the patient did not intend to continue her treatment. As a result, there are no follow‐up intraoral photographs, periapical radiographs or cone beam computed tomography images.

In conclusions, chloroform should be used with caution according to the patient's condition to prevent its potential consequences. Also, the case highlights the need for careful consideration of pain in patients subjected to chloroform during dental procedures. It is crucial to exercise caution, avoiding needle locking and excessive pressure, to minimize the risk of adverse reactions. These precautionary measures play a pivotal role in ensuring the safe and effective use of chloroform in dental practices.

## AUTHOR CONTRIBUTIONS

**Zahra Sadat Modarresi:** Conceptualization; data curation; investigation; methodology; project administration; resources; validation; visualization; writing – original draft; writing – review and editing. **Sahar Shafagh:** Investigation; resources; writing – original draft; writing – review and editing. **Mehrzad Kaviani:** Writing – original draft; writing – review and editing. **Mojtaba Salehi Karizmeh:** Conceptualization; data curation; investigation; project administration; software; supervision; writing – original draft; writing – review and editing.

## FUNDING INFORMATION

The authors received no financial support for this research.

## CONFLICT OF INTEREST STATEMENT

The authors declare that they have no conflict of interest.

## CONSENT

Written informed consent was obtained from the patient to publish this report in accordance with the journal's patient consent policy.

## Data Availability

The data that support the findings of this study are available on request from the corresponding author. The data are not publicly available due to privacy or ethical restrictions.
